# Robust Automated Detection of Microstructural White Matter Degeneration in Alzheimer’s Disease Using Machine Learning Classification of Multicenter DTI Data

**DOI:** 10.1371/journal.pone.0064925

**Published:** 2013-05-31

**Authors:** Martin Dyrba, Michael Ewers, Martin Wegrzyn, Ingo Kilimann, Claudia Plant, Annahita Oswald, Thomas Meindl, Michela Pievani, Arun L. W. Bokde, Andreas Fellgiebel, Massimo Filippi, Harald Hampel, Stefan Klöppel, Karlheinz Hauenstein, Thomas Kirste, Stefan J. Teipel, the EDSD study group

**Affiliations:** 1 German Center for Neurodegenerative Diseases (DZNE), Rostock, Germany; 2 Institute for Stroke and Dementia Research (ISD), Ludwig Maximilian University, Munich, Germany; 3 Institute of Scientific Computing, Helmholtz Zentrum München – German Research Center for Environmental Health, Neuherberg, Germany; 4 Department for Database Systems, Technische Universität München, Munich, Germany; 5 Institute for Informatics, Ludwig Maximilian University, Munich, Germany; 6 Institute for Clinical Radiology, Department of MRI, Ludwig Maximilian University, Munich, Germany; 7 Laboratory of Epidemiology, Neuroimaging and Telemedicine (LENITEM), IRCCS Centro San Giovanni di Dio FBF, Brescia, Italy; 8 Cognitive Systems Group, Discipline of Psychiatry, School of Medicine, Trinity College Dublin, Dublin, Ireland; 9 Trinity College Institute of Neuroscience (TCIN), Trinity College Dublin, Dublin, Ireland; 10 Department of Psychiatry, University Medical Center Mainz, Mainz, Germany; 11 Neuroimaging Research Unit, Institute of Experimental Neurology, Division of Neuroscience, Scientific Institute and University Vita-Salute San Raffaele, Milan, Italy; 12 Department of Psychiatry, Psychosomatic Medicine and Psychotherapy, Goethe University, Frankfurt, Germany; 13 Freiburg Brain Imaging, Department of Psychiatry and Psychotherapy & Department of Neurology, University Medical Center Freiburg, Freiburg, Germany; 14 Department of Radiology, University of Rostock, Rostock, Germany; 15 Mobile Multimedia Information Systems Group (MMIS), University of Rostock, Rostock, Germany; 16 Clinic for Psychosomatic and Psychotherapeutic Medicine, University of Rostock, Rostock, Germany; University of Maryland, College Park, United States of America

## Abstract

Diffusion tensor imaging (DTI) based assessment of white matter fiber tract integrity can support the diagnosis of Alzheimer’s disease (AD). The use of DTI as a biomarker, however, depends on its applicability in a multicenter setting accounting for effects of different MRI scanners. We applied multivariate machine learning (ML) to a large multicenter sample from the recently created framework of the European DTI study on Dementia (EDSD). We hypothesized that ML approaches may amend effects of multicenter acquisition. We included a sample of 137 patients with clinically probable AD (MMSE 20.6±5.3) and 143 healthy elderly controls, scanned in nine different scanners. For diagnostic classification we used the DTI indices fractional anisotropy (FA) and mean diffusivity (MD) and, for comparison, gray matter and white matter density maps from anatomical MRI. Data were classified using a Support Vector Machine (SVM) and a Naïve Bayes (NB) classifier. We used two cross-validation approaches, (i) test and training samples randomly drawn from the entire data set (pooled cross-validation) and (ii) data from each scanner as test set, and the data from the remaining scanners as training set (scanner-specific cross-validation). In the pooled cross-validation, SVM achieved an accuracy of 80% for FA and 83% for MD. Accuracies for NB were significantly lower, ranging between 68% and 75%. Removing variance components arising from scanners using principal component analysis did not significantly change the classification results for both classifiers. For the scanner-specific cross-validation, the classification accuracy was reduced for both SVM and NB. After mean correction, classification accuracy reached a level comparable to the results obtained from the pooled cross-validation. Our findings support the notion that machine learning classification allows robust classification of DTI data sets arising from multiple scanners, even if a new data set comes from a scanner that was not part of the training sample.

## Introduction

The newly established diagnostic criteria for Alzheimer’s disease (AD) have stressed the detection of biological markers of disease for early diagnosis, even before the onset of dementia [1], [2]. Among those biomarkers are MRI derived measures of regional brain atrophy. A promising new imaging marker of AD are measures of structural disconnection using diffusion tensor imaging (DTI), consistent with the pathogenetically early involvement of axonal structures in AD [3]. DTI allows the reconstruction of the main directions of diffusion [4]. From DTI we can derive scalar indices of anisotropic diffusion, the most widely used being the fractional anisotropy (FA) and mean diffusivity (MD) as measures of microstructural white matter (WM) integrity. Reduced FA or increased MD indicate impaired WM fiber tract integrity [5], [6]. Previous studies have found a significant decline of fiber tract integrity in posterior cingulate, corpus callosum, temporal lobe and parietal lobe WM in AD [7], [8], [9], [10], [11], [12], [13], [14], [15], [16], [17]. In addition, multivariate voxel-based approaches to detect changes in structural connectivity within the whole WM showed a decline of structural network connectivity in AD dementia and even prodromal AD [17], [18], [19], [20]. These results agree with the notion that changes in microstructural integrity of fiber tracts subserving structural connectivity would precede the decline of neuronal density in gray matter areas such as the hippocampus [21].

Establishing DTI as an imaging marker of AD requires studying its diagnostic performance in large samples across multiple sites. Only few studies have investigated multicenter variability of DTI derived measures of fiber tract integrity [22], [23]. A recent study has shown that multicenter variability is about 50% higher in DTI derived FA measures compared to classical MRI volumetric measures of GM [24]. The question is unresolved to what extent the multicenter variability compromises the clinical utility of DTI for the detection of AD dementia.

Machine learning (ML) approaches are particularly sensitive to distributed disease-specific changes observed in many human structural and functional imaging studies [18], [25], [26], [27]. They are designed to identify patterns in data that differentiate between several classes. In the most basic approach, the univariate Naïve Bayes (NB) classifier [28], a group comparison of the intensity values is performed for each voxel separately, and the classification result is derived from the most likely class. Although NB relies on the naïve assumption of statistical independence of the features, previous results, e.g. Plant et al. [27], showed that NB performs well for the discrimination of AD patients from healthy controls even if the assumption of statistical independence of different features is violated. More advanced, multivariate approaches rely on aggregations of features for class separation. The Support Vector Machine (SVM) classifier [29], [30] has been successfully applied in several AD imaging studies, e.g. Klöppel et al. [31], Cuinget et al. [32], Abdulkadir et al. [33], Plant et al. [27] and Graña et al. [19], showing highly accurate results. For applications in future diagnostic expert systems ML algorithms must be robust and stable to work with data recorded across different scanners. The potential diagnostic accuracy of ML algorithms with DTI data gathered across different scanners, with different field strengths and different acquisition schemes, has not yet been investigated.

Within the European DTI Study on Dementia (EDSD) we have collected data of more than 330 subjects from ten scanners located at nine sites. Based on this data set, we aimed to assess the accuracy of ML classifiers for the automated detection of AD. We compared the diagnostic accuracy between the univariate NB classifier as baseline and the multivariate SVM. We used two complementary cross-validation approaches: first, we draw the test and training sets from the entire sample, second, we used the data from each single scanner as test set after learning with the data from the other scanners to validate the generalizability of our approach. We compared classification accuracies of NB and SVM before and after principal component analysis (PCA) and mean correction of the scans to reduce between scanner variability. We expected that the SVM ML algorithm would be robust against scanner effects and more accurate than the massive univariate NB classification approach, and that removal of scanner variance would be more relevant in the scanner-based validation than the pooled data validation approach. The findings of our study will be informative for the development of radiological expert systems geared towards the early detection of AD related neuronal disconnection.

## Materials and Methods

### Data Acquisition

The data were retrospectively identified from the European DTI Study on Dementia (EDSD), a newly established framework of nine European centers: Amsterdam (Netherlands), Brescia (Italy), Dublin (Ireland), Frankfurt (Germany), Freiburg (Germany), Milano (Italy), Mainz (Germany), Munich (Germany), and Rostock (Germany), with one center including data from two different MRI scanners.

At present (October 2012), the data set includes 335 DTI and 335 MRI scans from patients with AD and healthy elderly subjects. Written informed consent was provided by all subjects or their representatives. The study was approved by local ethics committees at each of the participating centers, i.e. (i) the ethics committee of the medical faculty of the Ludwig-Maximilian-University, Munich, (ii) the ethics committee of the IRCCS San Giovanni di Dio FBF, Brescia, (iii) the Faculty Research Ethics Committee, Faculty of Health Sciences, Trinity College Dublin, (iv) the ethics committee at the Landesärztekammer Rheinland-Pfalz, Mainz, (v) the ethics committee of San Raffaele Hospital, Milan, (vi) the ethics committee of the faculty of medicine of the Goethe University, Frankfurt, (vii) the ethics committee of the University Medical Center, Freiburg, and (viii) the ethics committee of the medical faculty of the University of Rostock. Due to susceptibility artifacts in the DTI data, all data from one center (N = 38) had to be excluded from further analysis. Additionally, 13 of 26 DTI scans, 2 of 35 DTI scans and 1 of 30 DTI scans from three other centers were excluded due to prominent artifacts in the data which were either caused by folding, high-frequency inferences, an incorrectly set inversion time or heavy movement artifacts. Another DTI scan had to be excluded due to imperfect normalization of the DTI data during preprocessing.

After preprocessing 280 DTI and MRI scans were retained for the analysis derived from eight centers representing nine MRI scanners. The data were derived from 137 patients with clinically probable AD according to NINCDS-ADRCA criteria [34] and 143 healthy elderly control subjects. All participants were free of any significant neurological, psychiatric or medical condition (except for AD in patients), in particular cerebrovascular apoplexy, vascular dementia, depression, subclinical hypothyroidism as well as substance abuse. Healthy controls were required to have no cognitive complaints and scored within one standard deviation of the age and education adjusted norm in all subtests of the Consortium to Establish a Registry of Alzheimer’s Disease (CERAD) cognitive battery [35]. Patients were significantly older and had less years of education than the controls (Table 1). Gender was not different between groups (Table 1). As expected, MMSE scores [36] were significantly lower in AD patients compared to controls, with the patients ranging in the mild to moderate stages of dementia [36]. The number of subjects per scanner ranged between 13 and 46 with a median of 29 (Table 2).

**Table 1:** Demographic data and MMSE of the subjects.

	AD	controls
No. of subjects (women)^1^	137 (79)	143 (72)
Age (SD) in years^2^	72.5 (8.3)	69.2 (5.9)
MMSE (SD)^3^	20.6 (5.3)	28.8 (1.1)
Years of education (SD)^4^	10.2 (3.3)	13.1 (3.8)

1 not significantly different between groups, *χ* ^2^ (1) = 1.5, *p* = 0.22.

2 significantly different between groups, *t* (278) = 3.92, *p*<0.001.

3 significantly different between groups, Mann-Whitney *U* = 263, *p*<0.001.

4 significantly different between groups, *t* (271) = −6.7, *p*<0.001.

Abbreviations: SD, standard deviation; MMSE, mini-mental state examination; AD, Alzheimer’s disease.

**Table 2:** Scan parameters for DTI and number of subjects per scanner.

Center	Scanner	Tesla	TR	TE	gradients	b-values	voxel size [mm]	Gap [%]	iPAT	averages	number of subjects (AD)
I	Allegra	3.0	5000	118	30	0; 1000	2×2×6	20	2	1	33 (17)
II	Achieva	3.0	12396	52	15	0; 800	2×2×2	0	2	2	29 (9)
III	Trio	3.0	146	100	60	0; 1000	2×2×2	0	2	1	24 (16)
IV	Trio	3.0	11800	94	61	0; 1000	2×2×2	0	2	1	13 (4)
V	Sonata	1.5	8000	105	6	0; 1000	2×2×3	0	2	1	31 (18)
VI	Avanto	1.5	6500	95	12	0; 1000	2×2×2.5	0	2	3	29 (15)
VII	Trio	3.0	9300	102	12	0; 1000	2×2×2	0	2	4	46 (26)
VIII	Avanto	1.5	5100	85	30	0; 1000	2×2×2.4	20	2	3	40 (15)
IX	Verio	3.0	8200	93	20	0; 1000	2×2×2	0	2	3	35 (20)

Abbreviations: TR, repetition time; TE, echo time; iPAT, integrated parallel imaging techniques; AD, Alzheimer’s disease.

### Data Preprocessing

Preprocessing of DTI data was performed using the diffusion toolbox of FSL (Version 4.1, FMRIB, Oxford, UK, http://www.fmrib.ox.ac.uk/fsl/) [37]. Preprocessing included corrections for eddy currents and head motion, skull stripping with the Brain Extraction Tool and fitting of diffusion tensors to the data with DTIfit. Deformation-based analysis of MPRAGE data and of the FA and MD maps was performed using SPM8 (Wellcome Trust Centre for Neuroimaging, London, UK, http://www.fil.ion.ucl.ac.uk/spm/) implemented in Matlab 7 (Mathworks, Natwick). The images in native space were manually aligned to set the anterior commissure as the origin of coordinate system and then FA and MD maps were affinely aligned to the corresponding MPRAGE scans.

For spatial normalization, the VBM8 toolbox (Version 414, http://dbm.neuro.uni-jena.de/vbm8/) [38] implemented in SPM8 was used to create a customized DARTEL template. To include an equally large sample from every scanner, we created the template out of N = 54 images, randomly selecting six scans (three AD patients and three healthy controls) from each of the nine scanners. The resulting template was used for high-dimensional DARTEL normalization of the MPRAGE scans as implemented in VBM8. Images were segmented into gray matter (GM) and white matter (WM) and transformed to MNI space applying modulation for non-linear components only. The Deformation fields derived from this step were applied to the spatially coregistered FA and MD maps, without modulation. To exclude all voxels outside the WM of the FA and MD maps, we used a binary WM mask based on the average WM image derived from the random sample of N = 54 normalized images described above. Additionally, we created a corresponding binary GM mask following the same procedure. The GM and WM segments as well as the masked FA and MD maps in MNI space were smoothed using an 8 mm full width at half maximum (FWHM) isotropic Gaussian kernel. After smoothing, all scans were again masked with the WM or GM mask, respectively, to restrict the subsequent analysis to be performed based on the voxels within the corresponding areas, only. Without additional masking after smoothing our subsequent analysis detected group differences in areas outside the respective tissues, e.g. in the ventricles. These artifacts were caused by imperfect smoothing at the segment or tissue borders.

### Classification Methods

**Figure 1:**
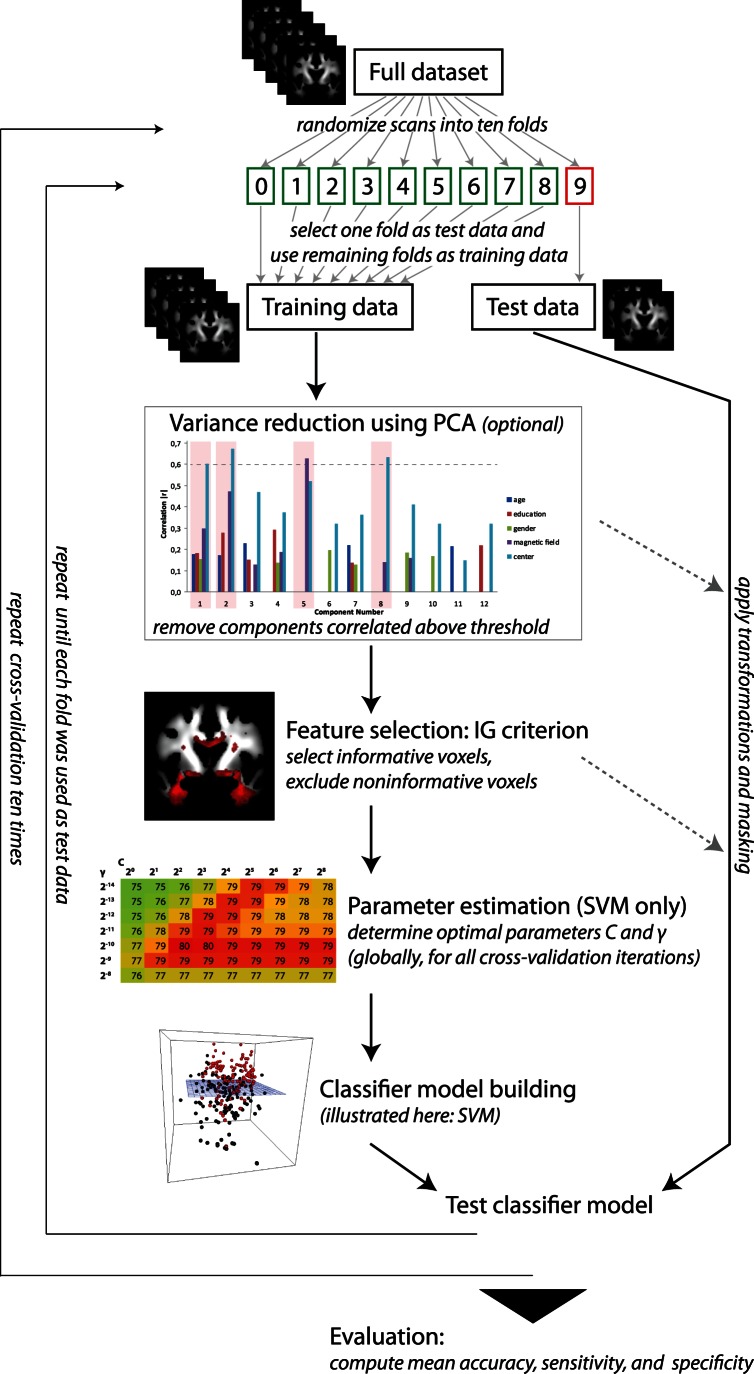
Flow chart of the ML analysis.

For classification, the four modalities gray matter density (GMD), white matter density (WMD), WM FA and WM MD were processed separately. For learning and classification we used the approach suggested in Plant et al. [27] and the WEKA machine learning toolkit (Version 3.6.6, http://www.cs.waikato.ac.nz/ml/weka/) [39]. The learning and classification process is illustrated in Figure 1 and involves three main steps: (i) feature selection, (ii) learning and classification, and (iii) evaluation. In order to estimate the performance of our methods objectively we used two cross-validation approaches: first, we pooled all data and divided them into a training set and a test set using the tenfold cross-validation technique (pooled cross-validation). All scans from the 280 subjects were randomized and stratified with respect to the diagnosis into ten folds using WEKA. For each iteration one fold was used as test data to evaluate the prediction accuracy and the remaining folds were used as training data. We repeated the tenfold cross-validation ten times for a more general performance estimation of the classifier. Second, we used the data from each single scanner as test set and the data from the remaining scanners as training set (scanner-specific cross-validation). This allowed us to evaluate the generalizability of our methods by simulating that they were applied to data from a new scanner.

### Feature Selection

The scans originally contained more than two million voxels. After image segmentation and masking, WM and GM tissue maps included 236,389 and 254,799 voxels, respectively. To reduce the computation time and memory space needed for data processing, and to improve the ML algorithm performance, the number of features (i.e. voxels) was further reduced: Following Plant’s approach [27], features that did not contribute any information to the separation of the data were excluded using the entropy-based information gain (IG) criterion [40], [41]. The IG is an information theoretic value describing how much a feature, in our case a certain voxel, contributes to the separation of the data. We decided to use the IG, because it has successfully been employed in a previous study on structural MRI [27], allowing for comparison of performance with the previous data set.

#### Notations

Given are a discrete set of classes, i.e. the diagnosis, *C = {AD, HC}* and a data set *DS* consisting of MRI scans of *n* subjects *s_1_, …, s_n_* labeled with a class. For each subject we have an MR image that is represented by a feature vector composed of *d* voxels *v_1_, …, v_d_*. We refer to the class label of subject *s_i_* by *s_i_.c*.

Entropy of the class distribution. The entropy of the class distribution *H(C)* is defined as 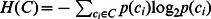, where *p(c_i_)* denotes the probability of class *c_i_*, i.e. 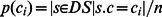. *H(C)* corresponds to the required amount of bits needed to identify the class of an unknown subject and scales between zero and one. In case of two classes and if the number of subjects per class is equal for both classes, *H(C) = 1*. In case of unbalanced class sizes the entropy of the class distribution is smaller than one and approaches zero if there are much more instances of one class than of the other class.

Information gain of a voxel. The information gain *IG(v_i_) = H(C)–H(C|v_i_)* of a voxel *v_i_* describes the difference of the entropy of the class distribution H(C) and the additional amount of information provided by *v_i_* on the class, which is noted as the conditional entropy *H(C|v_i_)*. In case of two classes the information gain scales between zero and one. A value of zero means that the feature does not contribute any information to the differentiation of the data. In contrast, a value of one indicates that the class labels of all subjects can be derived from the corresponding voxel without any errors. To compute the conditional entropy, features with continuous values need to be discretized using the algorithm of Fayyad and Irani [42]. This method divides the attribute range into class pure intervals using a criterion based on the minimum description length principle to determine the optimal number and location of the cut points (for a more extensive description of this algorithm see Section 7.2 in Witten and Frank [39]).

After computing the IG for each voxel of the training scans a density-based clustering algorithm [27], [43] was applied to remove noise and to only retain groups of more than seven contiguous voxels. The value of seven is derived from a voxel – which can be seen as a cube – and its six direct neighbors. The resulting clusters define the area of interest which was used to mask both, the training data and the test data.

### Learning and Classification

For classification we used two different types of classifiers: (i) a multivariate Support Vector Machine (SVM) [29], [30] with a radial basis function (RBF) kernel and (ii) a univariate Naïve Bayes (NB) classifier [28] as baseline. SVM performed highly accurately in former studies, e.g. in Klöppel et al. [31], Plant et al. [27], Cuinget et al. [32], and Graña et al. [19]. In contrast, NB is simple and efficient but relies on the naïve assumption of statistical independence of the features. Under this assumption this algorithm is statistically optimal regarding the minimal error rate. Preceding results, e.g. Plant et al. [27], showed that NB performs well for the detection of AD vs. HC even if this assumption of statistical independence of different features is not correct.

For the SVM we needed to define two parameters including the complexity or cost constant *C* and the radial basis function kernel width (*γ*>0). The parameter >0 determines the trade-off between margin maximization and training error minimization for the soft margin SVM. To estimate suitable values for *C* and *γ* we used a grid search in the range of *C = *2^−3^, 2^−2^, …, 2^8^ and *γ = *2^−14^, 2^−13^, …, 2^−1^ which we performed for each modality separately. Using the same parameter space for each modality is appropriate as the range of every input feature was rescaled to be between zero and one before applying the SVM algorithm. Due to high computational costs of SVM parameter estimation we used a two-step approach: First, we computed the accuracy of the SVM classifier for the whole range of parameters with only two arbitrarily selected folds. Then, we selected a smaller area for the parameter range in which we repeated the parameter estimation process for all of the other folds. For the parameter estimation we performed an internal fourfold cross-validation for the training data. Thus, the test data were not used for parameter selection. The parameters which gave the best average results for all repetitions were applied for the final classification and validation process. For the NB classifier we assumed a Gaussian distribution of the features for both groups (AD and HC). The distribution parameters were estimated based on the training data using the maximum likelihood method.

For feature selection, classifier model building, and parameter estimation, we used the training data, only. This method ensured that the test data was strictly separated from these steps and solely used to evaluate the prediction of previously generated models.

### Evaluation

As results we report the mean accuracy, sensitivity and specificity. The accuracy was defined as *accuracy* = (|TP|+|TN|)/n where |TP| is the number of true positives, |TN| is the number of true negatives and n is the total number of subjects. Following a common convention, we defined correctly classified patients with AD as true positives. The sensitivity and the specificity measure the ability of a classifier to identify positive and negative instances, i.e. *sensitivity* = |TP|/(|TP|+|FN|), *specificity* = |TN|/(|TN|+|FP|), where |FN| and |FP| are the number of false negative and false positive instances, respectively. As it is an open research problem to estimate the classification error we provide the 2.5 and 97.5 percentiles of our results as 95% confidence interval.

### Visualization

To assess which voxels contributed most to the separation of the data we performed sensitivity analysis [44], [45] of the learned SVM models. In contrast to previous studies, e.g. Cuinget et al. [32] and Klöppel et al. [31], we could not use the weight vector of the learned linear SVM model to visualize which voxels were used to separate the data. This approach is only applicable for the linear kernel SVM for which the classifier model can be simplified to a linear combination of features, i.e. voxel intensity values [46], [47]. Instead, we used a nonlinear combination of voxels (the Gaussian radial basis function kernel) for which an approximate visualization technique, called sensitivity analysis, is available. Sensitivity analysis is a heuristic which assesses the relative importance of a single voxel for classification. Thus, it provides a relative measure of how much the value of a certain voxel influences the outcome of the learned SVM model [44]. To calculate the sensitivity maps we used the Matlab script of Rasmussen, which is freely available at http://petermondrup.com/?page_id=127.

In order to determine the most informative anatomical locations from the sensitivity maps, we computed the mean image from all 100 maps (each run and fold). As different areas within the mean sensitivity maps were contiguous and could not be clearly separated from each other, we used a custom algorithm that identified the location of the highest sensitivity values within a predefined range of adjacent voxels. Then, the anatomical location information was obtained with the Talairach Daemon software available at http://www.talairach.org after MNI to Talairach coordinate transformation using the icbm2tal script of Lancaster et al. [48] available at http://brainmap.org/icbm2tal. Finally, we manually verified the results by comparing them with those reported by the FSL atlasquery tool (FSL Version 4.1, FMRIB, Oxford, UK, http://www.fmrib.ox.ac.uk/fsl/) [37] and the printed Talairach atlas [49].

### Variance Reduction

In order to reduce the variance introduced by different scanners and other confounding factors, for the pooled cross-validation approach we used principal component analysis (PCA). This method has been used for dimension reduction in previous neuroimaging studies, e.g. in Teipel et al. [18] and Zuendorf et al. [50]. We hypothesized that PCA will capture disease related variance but also systematic noise, e.g. between-scanner differences, which can then be removed to improve the classification accuracy.

We integrated variance reduction after randomizing the scans into training sets and test sets and before selecting the features and building the classifier model (see Figure 1). Again, for all steps we used solely the training data to calculate the parameters and subsequently applied the transformations to both the training data and the test data. First, we standardized the training data using voxel-wise z-score transformation: *z_i,k_ = (x_i,k_–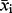)/s_i_*, where *x_i,k_* is the value of voxel *i* in scan *k*, 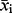 is the mean value of voxel *i* across all scans and *s_i_* is the standard-deviation of voxel *i* across all scans. Then, we computed the eigenvalues ***λ*** *_i_* and eigenimages ***v*** *_i_* of the covariance matrix ***X*** *_Training_* ***X*** *_Training_^T^* of the training data matrix ***X*** *_Training_* using PCA. We projected the original images into the component space ***Y*** *_Training_* = ***V*** *_Training_* ***^T^ X*** *_Training_* using the eigenimages as new basis of the coordinate system ***V*** *_Training_ = (* ***v*** *_1_,* ***v*** *_2_ …,* ***v*** *_n_)*. Subsequently, we correlated the eigenimage scores contained in the new data vectors ***Y*** *_Training_ = (* ***y*** *_1_,* ***y*** *_2_, …,* ***y*** *_n_)^T^* with confounding factors, i.e. the subjects’ age, gender and duration of education, the magnetic field strength of the scanner and the center in which the subject was scanned, using Pearson’s correlation. We removed the eigenimages for which the correlation with the scores superseded a prespecified threshold for at least one of the confounding factors by zeroing the corresponding eigenimages. For instance, if the first two eigenimages are removed, the new partial basis ***V*** *_Training,partial_ = (* ***v*** *_0_,* ***v*** *_0_,* ***v*** *_3_, …,* ***v*** *_n_)* will contain two zero-vectors ***v*** *_0_* instead of the first two eigenimages ***v*** *_1_* and ***v*** *_2_*. Finally, we projected the cleaned training images back into the original image space ***X*** *_Training,cleaned_ =* ***V*** *_Training,partial_* ***Y*** *_Training_* using the partial basis ***V*** *_Training,partial_*. For the test data we first applied the same scaling parameters as determined previously for the z-score transformation of the training data. Then, we also projected the test data matrix ***X*** *_Test_* into the component space using the full basis ***Y*** *_Test_ =* ***V*** *_Training_^T^* ***X*** *_Test_* and reprojected it into the original image space using the partial basis ***X*** *_Test,cleaned_ =* ***V*** *_Training,partial_* ***Y*** *_Test_*. Subsequently, we repeated the full feature selection and classification procedure as described above.

No unique criterion exists to define if two components are highly correlated. As a common rule of thumb Cohen [51] (p.77ff) suggested |*r*|>0.5 as high correlation so that *r^2^* = 0.25 of the variance is accounted for by the explanatory variable. In contrast, Hardy and Bryman [52] (p.27f) defined the correlation in the range of 0.7 to 0.9 as strong, while correlations in the range of 0.4 to 0.6 were rated as being moderate. Here, we first determined a correlation threshold from the highest occurring values of the histogram of correlations and subsequently reduced this threshold by steps of 0.1 to compare the results. For comparison, we additionally computed the correlation of diagnosis with the components.

PCA and similar variance reducing approaches need the complete data set to estimate the optimal model parameters. In order to evaluate the performance of the ML algorithms for the scanner-specific cross-validation, we wanted the test data to be excluded from the parameter estimation process and the learning step. Under the assumption that different scanners and scan parameters introduce independent variance, it is highly probable that a certain bias will remain in the test dataset after applying the variance reduction. Therefore, we calculated a mean image for each scanner by averaging the voxel values across all healthy subjects. The mean images were then used for voxel-wise mean centering, that means to subtract the corresponding mean image from every scan: *x^*^_i,k_ = x_i,k_–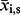
*, where *x_i,k_* is the original value of voxel *i* in scan *k*, 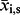 is the mean value of voxel *i* across all scans from healthy subjects scanned in scanner *s*. We did not apply a full z-score transformation or other rescaling operation because the low number of eight or nine healthy subjects in some centers would have introduced an additional bias.

## Results

### Feature Selection

For the WMD maps and DTI data the IG values ranged between 0 and 0.25, for the GMD maps between 0 and 0.5. We empirically determined 0.05 as threshold from the histogram of the IG values and clustered the IG maps with this threshold. Figure S1 shows the clusters of informative voxels derived from the averaged IG maps for each of the modalities. Masking with the clustered IG maps reduced the number of voxels to around 26*10^3^ for FA maps, which corresponds to 11% of the number of voxels of the WM tissue mask (Table 3). For MD maps approximately 128*10^3^ voxels (54%) were selected, for WMD 41*10^3^ voxels (17%) and for GMD 181*10^3^ voxels (71%). Masking of the corresponding scanner-based folds kept roughly the same number of voxels (Table 4) except for the FA maps. For those, approximately 11*10^3^ voxels (5%) and 49*10^3^ voxels (21%) were selected in the validation process using the original scans or the mean corrected scans, respectively.

**Table 3:** SVM classification results for the original and PCA variance reduced data (pooled cross-validation).

Modality		Accuracy [%]	Sensitivity [%]	Specificity [%]	No. of features [10^3^ voxels]	Reduced variance
FA	original	80.3 [66.0, 94.7]	78.8 [57.1, 96.6]	81.9 [64.3, 100.0]	26 (11%)	–
	reduced |*r*|>0.6	81.8 [71.4, 100.0]	78.0 [57.1, 100.0]	85.5 [65.4, 100.0]	23 (10%)	29%
	reduced |*r*|>0.5	79.9 [66.0, 89.5]	74.5 [53.4, 96.6]	85.1[64.3, 100.0]	22 (9%)	46%
	reduced |*r*|>0.4	78.3 [62.5, 89.3]	74.4 [50.0, 100.0]	82.0 [57.1, 100.0]	21 (9%)	58%
MD	original	83.3 [69.1, 96.4]	79.6 [57.1, 100.0]	86.9 [71.4, 100.0]	128 (54%)	–
	reduced |*r*|>0.6	83.4 [70.1, 94.7]	75.9 [55.4, 92.9]	90.7 [71.4, 100.0]	67 (28%)	31%
	reduced |*r*|>0.5	82.9 [71.4, 93.0]	74.8 [57.1, 92.9]	90.6 [75.8, 100.0]	49 (21%)	56%
	reduced |*r*|>0.4	82.2 [67.9, 94.7]	74.2 [51.8, 92.9]	89.8 [66.7, 100.0]	43 (18%)	63%
WMD	original	82.7 [67.9, 96.4]	77.9 [55.4, 92.9]	87.4 [71.4, 100.0]	41 (17%)	–
	reduced |*r*|>0.6	–	–	–	–	–
	reduced |*r*|>0.5	81.1 [66.0, 93.0]	74.2 [50.0, 92.9]	87.8 [65.4, 100.0]	60 (25%)	23%
	reduced |*r*|>0.4	79.1 [64.3, 92.9]	72.8 [51.8, 92.9]	85.2 [64.3, 100.0]	53 (22%)	45%
GMD	original	89.3 [78.6, 100.0]	87.4 [69.2, 100.0]	91.2 [72.3, 100.0]	182 (71%)	–
	reduced |*r*|>0.6	–	–	–	–	–
	reduced *|r*|>0.5	–	–	–	–	–
	reduced |*r*|>0.4	74.6 [57.1, 89.3]	66.3 [40.5, 85.7]	82.7 [64.3, 100.0]	20 (8%)	32%

For each modality the average number of informative voxels is provided and in parentheses the proportion compared to the respective tissue masks is presented. In the last column the removed variance proportion is given.

Abbreviations: FA, fractional anisotropy; MD, mean diffusivity; WMD, white matter density; GMD, gray matter density.

**Table 4:** Cross-validation results using the data of each scanner as fold.

Modality		ML algorithm	Accuracy [%]	Sensitivity [%]	Specificity [%]	No. of features [10^3^ voxels]
FA	original	SVM	73.8 [57.8, 86.0]	73.0 [13.1, 94.0]	70.4 [19.3, 98.0]	11 (5%)
		NB	69.4 [52.6, 88.6]	68.3 [19.0, 100.0]	70.5 [12.0, 100.0]	
	mean corrected	SVM	76.2 [60.5, 91.1]	65.0 [44.3, 97.8]	86.3 [52.0, 100.0]	49 (21%)
		NB	72.7 [63.8, 89.2]	66.7 [30.7, 93.8]	77.8 [62.5, 89.8]	
MD	original	SVM	63.6 [46.6, 82.3]	72.2 [25.3, 100.0]	58.5 [20.0, 97.5]	129 (55%)
		NB	68.0 [57.7, 89.0]	54.0 [26.0, 97.8]	81.4 [25.0, 100.0]	
	mean corrected	SVM	78.3 [67.0, 94.8]	60.8 [26.7, 93.1]	92.4 [80.3, 100.0]	157 (66%)
		NB	72.7 [58.1, 88.2]	63.3 [35.4, 94.0]	82.0 [33.8, 95.0]	
WMD	original	SVM	78.8 [58.7, 91.8]	72.6 [42.0, 98.7]	85.7 [47.0, 100.0]	42 (18%)
		NB	73.4 [58.8, 81.9]	67.4 [50.0, 87.6]	77.6 [57.7, 99.0]	
	mean corrected	SVM	85.4 [71.5, 98.6]	73.8 [43.3, 97.8]	96.7 [88.6, 100.0]	55 (23%)
		NB	73.0 [52.3, 83.6]	61.5 [31.3, 84.3]	84.5 [66.3, 98.8]	
GMD	original	SVM	82.4 [71.9, 97.5]	82.2 [52.0, 100.0]	84.1 [48.0, 100.0]	180 (71%)
		NB	69.9 [35.8, 91.4]	65.0 [0.8, 97.6]	78.0 [13.7, 100.0]	
	mean corrected	SVM	91.1 [82.7, 100.0]	84.0 [67.9, 100.0]	98.3 [95.0, 100.0]	200 (78%)
		NB	70.4 [61.0, 82.1]	67.1 [47.3, 91.7]	74.5 [58.2, 86.0]	

For each modality the average number of informative voxels is provided and in parentheses the proportion compared to the respective tissue masks is presented.

Abbreviations: FA, fractional anisotropy; MD, mean diffusivity; WMD, white matter density; GMD, gray matter density; ML, machine learning; SVM, Support Vector Machine; NB, Naïve Bayes.

### Classification

An overview of the classification results for the pooled cross-validation is given in Table 3 (SVM) and Table 5 (NB). In summary, we obtained a mean accuracy of 80.3% for FA and 83.3% for MD with the multivariate SVM classifier. We achieved 82.7% accuracy for WMD and 89.3% accuracy for GMD. The accuracies for the DTI indices were significantly smaller than those for the GMD maps with *p*<0.001 when we compared them across the ten repetitions (two-tailed paired t-test). For the mass-univariate NB classifier we achieved an average accuracy of 70.3% for FA, 69.7% for MD, 75.1% for WMD and 71.5% for GMD. These results were significantly lower than those of the SVM with *p*<0.001 when we compared the mean accuracies of the ten repetitions (two-tailed paired t-test). Each of the SVM classifier models concord for on average 70% of the subjects across all four modalities. In the pair-wise comparison up to 79% of the subjects were correctly identified by the SVM models for MD and GMD. Four percent of the subjects were additionally correctly identified by the DTI indices compared to GMD. In contrast, the GMD SVM model additionally identified ten to twelve percent of the subjects compared to each of the other modalities. For the NB classifier the classifier models concord in 41% of the subjects across all modalities. In the pair-wise comparison 51–57% were correctly classified by both classifier models while 14–20% of the subjects were correctly identified by either of the classifiers (approximately equally distributed). The results for the scanner-specific cross-validation are given in Table 4. For the SVM classifier we obtained a mean accuracy of 73.8% for the FA maps, 63.6% for MD, 78.8% for WMD, and 82.4% GMD. For the NB classifier we received 69.4 for FA, 68.0% for MD, 73.4% for WMD, and 69.9% for GMD.

**Table 5:** NB classification results for the original and PCA variance reduced data (pooled cross-validation).

Modality		Accuracy [%]	Sensitivity [%]	Specificity [%]
FA	original	70.4 [56.1, 84.0]	65.4 [42.9, 85.7]	75.1 [57.1, 93.3]
	reduced |*r*|>0.6	72.2 [54.3, 86.2]	72.7 [51.8, 92.9]	71.8 [42.9, 93.3]
	reduced |*r*|>0.5	71.1 [54.5, 85.7]	71.2 [50.0, 92.9]	71.0 [44.7, 92.9]
	reduced |*r*|>0.4	71.3 [53.6, 85.7]	70.0 [44.4, 92.9]	72.6 [46.3, 92.9]
MD	original	68.8 [53.6, 80.8]	50. 9 [28.6, 75.2]	85.9 [71.4, 100.0]
	reduced |*r*|>0.6	71.1 [54.3, 85.7]	55.6 [32.0, 78.6]	85.9 [64.3, 100.0]
	reduced |*r*|>0.5	68.6 [54.3, 80.8]	43.5 [16.0, 70.4]	92.7 [78.6, 100.0]
	reduced |*r*|>0.4	68.6 [55.3, 79.0]	44.5 [21.4, 71.4]	91.7 [74.8, 100.0]
WMD	original	74.7 [53.6, 89.5]	70.9 [40.5, 92.9]	78.4 [53.3, 100.0]
	reduced |*r*|>0.6	–	–	–
	reduced |*r*|>0.5	72.4[53.6, 91.2]	62.5 [35.7, 89.2]	82.0 [57.1, 100.0]
	reduced |*r*|>0.4	68.6 [57.1, 82.1]	57.0 [30.8, 78.6]	79.7 [58.5, 100.0]
GMD	original	69.9 [51.7, 84.0]	61.6 [35.7, 82.3]	78.1 [55.1, 92.9]
	reduced |*r*|>0.6	–	–	–
	reduced |*r*|>0.5	–	–	–
	reduced |*r*|>0.4	66.0 [42.9, 85.7]	63.3 [40.5, 85.7]	68.6 [40.0, 92.9]

Abbreviations: FA, fractional anisotropy; MD, mean diffusivity; WMD, white matter density; GMD, gray matter density.

### Visualization

**Figure 2:**
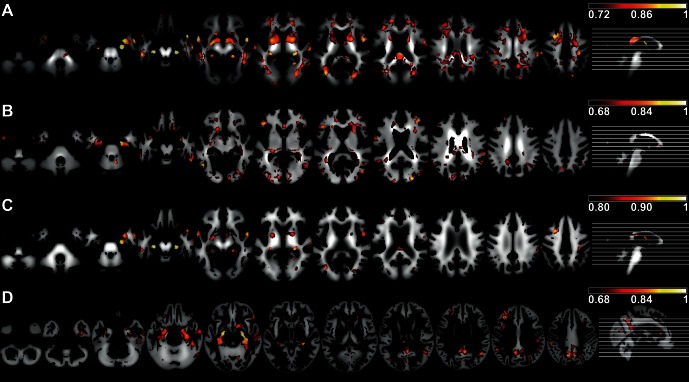
SVM sensitivity maps (upper 5% percentiles). Sensitivity maps for (A) FA, (B) MD, (C) WMD, and (D) GMD. The maps display the relative importance of each voxel for the classification decision, with white/yellow areas being more important than red areas. Preceding SVM classification, voxels that did not contribute any information to the group separation of AD and HC were masked out (IG criterion). The slices shown are: −46, −38, −28, −20, −10, −2, 8, 16, 26, 34, and 44 in MNI space.

Figure 2 and Figure S2 show the averaged sensitivity maps for each of the modalities. For easier interpretation, Figure 2 displays only the upper 5% percentiles of the sensitivity values. To scale sensitivity values, we first applied the natural logarithm to the sensitivity values and then rescaled them to be between zero and one. Table 6, Table 7, Table 8, and Table 9 report the anatomical locations of the twenty highest sensitivity values for each modality. We restricted these points to be at least 10.5 mm distant from each other. For FA, we found large areas with high sensitivity values in the corpus callosum and medial temporal lobes, including WM of the hippocampus, cingulate gyrus WM, precuneus, parietal, temporal, and prefrontal lobe WM, but also in a small cluster in the left precentral and postcentral gyrus WM and WM around the basal ganglia. For MD, high sensitivity values clustered within the lateral and medial temporal lobe WM, including parahippocampal gyrus and fusiform gyrus, prefrontal lobe WM, parietal and occipital WM. There was an additional cluster in the cerebellum For WMD, high sensitivity values occurred in prefrontal lobe, medial temporal lobe, corpus callosum and WM areas of the caudate nucleus and putamen. For GMD, high sensitivity values occurred in parieto-temporal association cortices, medial temporal lobe, including hippocampus and parahippocampal gyrus, and cingulate gyrus. Additionally, the putamen was involved. For all indices, brainstem, primary sensori-motor areas and the cerebellum were almost devoid of AD related changes.

**Table 6:** Anatomic areas of the twenty most informative voxels derived from the averaged SVM sensitivity maps for FA.

		Coordinates (mm)			
Region	Side	x	y	z	Sensitivity
Cuneus WM	L	−29	−65	14	1.00
Precentral gyrus WM	L	−17	−51	36	0.98
Parietal lobe WM	L	2	−18	23	0.98
Temporal lobe WM	R	32	−21	20	0.94
Parahippocampal gyrus WM	R	24	−23	−21	0.93
Uncus WM	L	−35	−2	−27	0.92
Parietal lobe WM	L	6	−5	24	0.92
Uncus WM	R	33	−6	−24	0.91
Parahippocampal gyrus WM	L	−38	−18	−14	0.90
Temporal lobe WM	L	−2	−6	14	0.90
Fornix	L	35	−48	2	0.89
Postcentral gyrus WM	L	−17	−29	27	0.88
Cingulate gyrus WM	R	12	20	18	0.87
Postcentral gyrus WM	L	−17	−11	29	0.87
Frontal lobe WM	L	−29	2	−12	0.87
Fusiform gyrus WM	L	48	−50	−14	0.85
Temporal lobe WM	L	−36	−65	−9	0.85
Corpus callosum	R	−38	−35	−5	0.84
Cuneus WM	L	−27	−39	20	0.84
Insula WM	R	27	−45	17	0.84

These points were restricted to be at least 10.5 mm distant from each other. The coordinates given are in MNI space. For easier interpretation, we first applied the natural logarithm to the sensitivity values and then rescaled them to be between zero and one.

Abbreviations: WM, white matter; L, left hemisphere; R, right hemisphere.

**Table 7:** Anatomic areas of the twenty most informative voxels derived from the averaged SVM sensitivity maps for MD.

		Coordinates (mm)			
Region	Side	x	y	z	Sensitivity
Inferior frontal gyrus WM	R	39	27	2	1.00
Parahippocampal gyrus WM	R	20	−11	−29	1.00
Middle occipital gyrus WM	R	24	−90	18	0.98
Fusiform gyrus WM	L	−48	−62	−12	0.97
Superior parietal lobule WM	L	−29	−62	54	0.96
Inferior temporal gyrus WM	R	60	−30	−24	0.95
Middle occipital gyrus WM	R	36	−89	11	0.95
Middle frontal gyrus WM	R	44	41	14	0.94
Inferior frontal gyrus WM	L	−45	30	−2	0.94
Cerebellum WM	R	8	−54	−27	0.93
Lingual gyrus WM	R	−33	−14	−8	0.92
Fusiform gyrus WM	R	30	−77	−14	0.91
Parahippocampal gyrus WM	L	−29	−8	−30	0.91
Putamen WM	L	−24	2	−6	0.91
Middle frontal gyrus WM	R	44	15	32	0.91
Inferior temporal gyrus WM	R	59	−51	−17	0.91
Putamen WM	L	−14	23	−8	0.91
Putamen WM	R	35	−20	−5	0.90
Superior temporal gyrus WM	L	−38	2	−18	0.90
Supramarginal gyrus WM	L	−57	−41	38	0.90

These points were restricted to be at least 10.5 mm distant from each other. The coordinates given are in MNI space. For easier interpretation, we first applied the natural logarithm to the sensitivity values and then rescaled them to be between zero and one.

Abbreviations: WM, white matter; L, left hemisphere; R, right hemisphere.

**Table 8:** Anatomic areas of the twenty most informative voxels derived from the averaged SVM sensitivity maps for WMD.

		Coordinates (mm)			
Region	Side	x	y	z	Sensitivity
Parahippocampal gyrus WM	R	24	−23	−24	1.00
Limbic lobe WM	R	29	−29	−6	0.99
Parahippocampal gyrus WM	L	−26	−24	−20	0.98
Middle temporal gyrus WM	R	56	−62	2	0.97
Middle frontal gyrus WM	L	−29	9	45	0.96
Inferior temporal gyrus WM	L	50	12	−18	0.95
Superior temporal gyrus WM	L	−59	2	−9	0.95
Superior temporal gyrus WM	R	56	3	−12	0.95
Middle occipital gyrus WM	L	−36	−75	6	0.95
Supramarginal gyrus WM	R	59	−42	33	0.95
Middle temporal gyrus WM	R	54	2	−27	0.94
Lingual gyrus WM	R	50	12	2	0.93
Middle temporal gyrus WM	R	63	−17	−17	0.93
Precentral gyrus WM	R	24	−51	2	0.93
Insula WM	R	51	0	6	0.92
Fornix	L	−2	−6	11	0.92
Inferior parietal Lobule WM	L	−60	−26	38	0.92
Cerebellum WM	R	27	−56	−54	0.90
Lentiform nucleus, lateral globus pallidus WM	R	23	−5	−14	0.90
Superior temporal gyrus WM	R	−56	−32	−18	0.90

These points were restricted to be at least 10.5 mm distant from each other. The coordinates given are in MNI space. For easier interpretation, we first applied the natural logarithm to the sensitivity values and then rescaled them to be between zero and one.

Abbreviations: WM, white matter; L, left hemisphere; R, right hemisphere.

**Table 9:** Anatomic areas of the twenty most informative voxels derived from the averaged SVM sensitivity maps for GMD.

			Coordinates (mm)			
Region	Brodmann area	Side	x	y	z	Sensitivity
Middle frontal gyrus	6	L	−26	−2	48	1.00
Caudate tail		L	−21	−9	−11	0.97
Lentiform nucleus, lateral globus pallidus		R	27	−15	−11	0.97
Precuneus	7	R	11	−60	39	0.97
Precuneus	7	L	−11	−60	42	0.94
Hippocampus		R	33	−30	−5	0.94
Precentral gyrus	9	L	−33	27	35	0.90
Posterior cingulate	23	R	8	−53	23	0.89
Thalamus, pulvinar		L	−5	−30	17	0.88
Superior temporal gyrus	21	R	63	−15	−8	0.88
Amygdala		L	−38	−24	−11	0.88
Supramarginal gyrus	40	L	−51	−47	33	0.87
Middle occipital gyrus	19	R	−36	−74	12	0.86
Middle occipital gyrus	19	L	33	−80	12	0.86
Middle temporal gyrus	39	R	47	−59	26	0.86
Uncus	20	R	36	−9	−35	0.86
Caudate head		R	18	30	−3	0.86
Precuneus	7	R	3	−45	53	0.84
Middle frontal gyrus	9	L	−30	44	32	0.83
Supramarginal gyrus	40	R	51	−47	36	0.83

These points were restricted to be at least 10.5 mm distant from each other. The coordinates given are in MNI space. For easier interpretation, we first applied the natural logarithm to the sensitivity values and then rescaled them to be between zero and one.

Abbreviations: L, left hemisphere; R, right hemisphere.

#### Variance reduction

**Figure 3:**
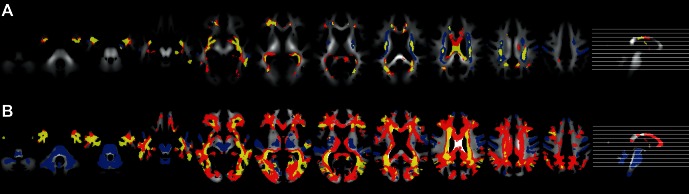
Comparison of informative voxel clusters. Comparison of the original cluster maps with the variance reduced ones for (A) FA and (B) MD. The slices shown are: −46, −38, −28, −20, −10, −2, 8, 16, 26, 34, and 44 in MNI space. Red – IG clusters of the original data, Blue – IG clusters of variance reduced data |r|>0.6, Yellow – overlap of both.

**Figure 4:**
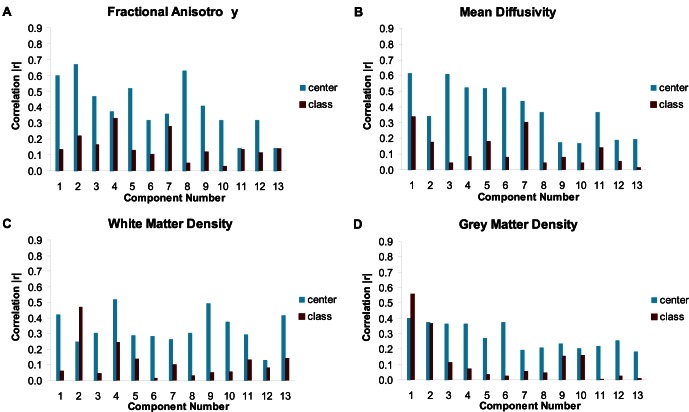
Principal components and correlated factors for a randomly selected training data set. Correlations for (A) FA, (B) MD, (C) WMD, and (D) GMD. The first thirteen components each explain at least 1% of the variance in the selected training data set.

For pooled cross-validation, the SVM classification results for the DTI data and the noise reduction approach using PCA are displayed in Table 3. For FA maps, the accuracy slightly but not significantly increased from 80.3% to 81.8% when highly correlated components (|*r*|>0.6) were removed. When reducing the correlation threshold to *|r|>0*.5 and |*r*|>0.4 the accuracy slightly decreased to 79.9% and 78.3%, respectively. For MD, the accuracy for the variance-reduced data set was 83.4% for |*r*|>0.6 and decreased slightly to 82.9% for |*r*|>0.5 and 82.2% for |*r*|>0.4, respectively. Figure 3 displays the IG clusters for FA and MD maps for the original data and variance reduced data sets (|*r*|>0.6) and their overlap. Figure 4 shows the correlation with the centers of the first thirteen principal components for a randomly selected training data set. Additionally, the correlations with the diagnosis are presented in Figure 4. Each of those components explains at least 1% of the variance in the selected training data set. For WMD, no components were correlated higher than |*r*|>0.6. The accuracy decreased from originally 82.7% to 81.1% for |*r*|>0.5 and 79.1% for |*r*|>0.4. Only few components of GMD were correlated above 0.4. The original accuracy of 89.3% dropped to 74.6% for |*r*|>0.4. In this case, the removed first component also carried a large amount of information about the diagnosis, it was correlated with diagnosis with |*r*| ≈ 0.6. The classification results for the NB classifier have a similar trend and are given in Table 5.

The results for the scanner-specific cross-validation after mean correction are given in Table 4. For the SVM classifier we obtained a mean accuracy of 76.2% for FA, 78.3% for MD, 85.4% WMD, and 91.1% for GMD. The accuracy for MD and the brain tissue density scans increased significantly compared to the original scans omitting mean correction with *p*<0.05 (two-tailed paired t-test). These values are slightly lower (DTI) or larger (brain tissue density) than the results obtained in the cross-validation with mixed folds but not statistically significant. For NB we received 72.7% for FA, 72.7% for MD, 73.0% for WMD, and 70.4% for GMD. The accuracies for the NB did not change significantly compared to the original scans, or compared to the cross-validation with mixed folds.

## Discussion

Our results indicate accurate and robust classification of patients with AD dementia and cognitively healthy elderly controls using ML based classification of multicenter DTI and MRI data. A classification accuracy of up to 91% for GM maps compares favorably with previous studies. Abdulkadir et al. [33] reported an accuracy of 87% for a multicenter GM data set comprising 417 subjects from the Alzheimer's Disease Neuroimaging Initiative (ADNI) database, while Cuinget et al. [32] obtained 81% sensitivity and 95% specificity for GM data from 299 subjects from the same database. Smaller studies yielded effects of similar magnitude. For example Klöppel et al. [31] achieved 87.5% accuracy for GM data from 68 subjects pooled from two centers and Plant et al. [27] 90% accuracy with 50 subjects from one single center.

For DTI parameters, we reached an accuracy of 82% (variance reduced) for the FA index and 83% for MD (mixed folds) and 76% and 78% (scanner-based folds), respectively. Graña et al. [19] reported a classification accuracy in the range of 97% to 100% for FA and 92% to 98% for MD for a monocenter DTI data set of 45 subjects. These earlier results seem quite optimistic and may reflect the relatively small number of participants compared to the large number of features.

Our SVM results for the DTI indices were significantly smaller than those for the GMD maps. This may partly be due to the clinically manifest disease stage of our patients which is typically associated with widespread cortical atrophy. Many studies investigated the dynamics of neuroimaging biomarkers during the progression of AD and found a significant correlations of either cortical atrophy measures or DTI based measures and MMSE [8], [53], [54], [55], [56], [57], [58]. But only few studies compared both anatomical MRI and DTI in this context [59], [60], [61]. In a meta-analysis, Clerx et al. [62] showed that in dementia stages of AD the effect sizes of volumetric medial temporal lobe atrophy measurements are superior to DTI derived measurements. In contrast, using regions of interest in the hippocampus, patients with amnestic mild cognitive impairment (MCI) showed a more accurate separation between MCI and healthy subjects using markers of diffusion anisotropy compared to hippocampus volume [62], [63], [64]. Therefore, even if structural connectivity was the more sensitive marker compared to volumetry in predementia stages of AD, this advantage may be offset by the higher multicenter variability of DTI measures at least when examining dementia stages of AD which are characterized by severe reductions of cerebral gray matter.

With 68% to 75% accuracy the results we obtained with the NB classifier were significantly lower than those of the SVM. These findings disagree with the high accuracy of NB based classification shown in a previous study with 50 subjects [27]. One possible explanation may be that we used multicenter data. Since the NB algorithm relies on differences in the distribution of voxel intensity values between diagnostic classes, additional variance introduced by the different scanners may have caused the reduction of classification accuracy. Secondly, as previously outlined by Rish [65], although the underlying assumption in NB of statistical independence of the features simplifies learning and training, it also leads to a loss of information which is contained in combinations of features. We assume that both factors explain the lower accuracy the of univariate NB classifier compared to multivariate SVM.

### Feature Selection

In our data-driven approach we used the IG criterion for voxel selection. The IG has the advantage over more widely used parameters, such as the t value or correlation coefficient, that it can directly be applied to multiclass data, which will be useful in future studies with additional diagnostic categories. In the study by Plant et al. [27] the IG values ranged between 0.1 and 0.6 for a combined GMD and WMD data set of 50 subjects. With our data, we expected lower values because pooling of data from different centers likely increased variability. We recorded IG values up to 0.5 for GMD and values up to 0.25 for the other modalities.

We used the feature selection step to exclude noninformative voxels. Although the empirically determined but possibly very liberal threshold of 0.05 kept a high number of voxels with a comparatively small amount of information, it enabled the multivariate SVM classifier to additionally exploit the information from those areas. Varying the IG threshold did not significantly increase the performance of the classifiers in our earlier empirical tests. Hence, we did not vary the minimum threshold for the IG values to further reduce the number of voxels to be used for classification. Alternatively, Graña et al. [19] used only the upper percentiles with different thresholds for voxel selection. In contrast, Klöppel et al. [31] and Cuinget et al. [32] omitted any feature selection step. We recommend preselecting informative voxels to reduce time and memory needed for the training and classification process.

Our approach recovered the typical anatomical areas that are involved in AD as shown in previous monocenter DTI and anatomical MRI studies [7], [8], [9], [10], [11], [12], [13], [14], [15], [16], [17], including medial temporal lobe, cortical association areas for GMD and the associated WM areas, including intracortically projecting fiber tracts such as the corpus callosum and fornix. These findings confirm the overall validity of our approach.

### Variance Reduction

When we performed PCA with pooled cross-validation we found several components that were highly correlated with scanner, particularly for FA and MD. These findings agree with results from a multicenter clinical and physical phantom study [24] suggesting 50% higher variability of FA values across centers compared to GMD. For FA, MD and WMD, classification accuracy from the SVM and regional distribution of group differences were relatively unaffected by removing variance components associated with age, gender and scanner. This suggests that the variance introduced by the scanner (and the other confounders) and the variance introduced by the diagnosis were largely independent from each other. The SVM algorithm and the NB classifier worked sufficiently robust to compensate the variance introduced by confounding factors so that the classification results remained almost unchanged after removing scanner-specific variance, as well as variance associated with age and gender.

For the scanner-specific cross-validation we used mean correction instead of PCA. Methods for variance reduction such as PCA or regression-based data correction need the complete data set to estimate the optimal model parameters. In order to evaluate the performance of the ML algorithms for new data objectively, we wanted the test data to be excluded from the parameter estimation process and the learning step. Given the assumption that different scanners and scan parameters introduce independent variance, it is highly probable that a certain bias will remain in the test dataset after applying the variance reduction. Therefore, we used the very basic approach of mean correction which we could apply to the data from each scanner, independently. Mean correction significantly increased the accuracy of the SVM classifier for MD and the tissue density scans. These results confirm that the indices obtained from DTI as well as the tissue density maps obtained from anatomical MRI depend on the scanner and the used scan parameters. The SVM algorithm seems to be highly sensitive to the bias introduced by the scanner and scan parameters to the DTI data – in case of the uncorrected data the accuracy drops to a level corresponding to the accuracy obtained using the mass-univariate NB classifier. In case of the pooled cross-validation the SVM algorithm was able to adapt its internal model to the higher variability of the scans during the learning phase. In contrast, in the scanner-specific cross-validation the data from one scanner were excluded from learning such that the SVM algorithm optimized its internal model using the data from the other scanners, only. Thus, the SVM sensitivity and specificity was highly depending on the ‘similarity’ of the test scans to the scans included in the learning data set. Our results for the mean corrected data show that this method is well suited to correct the bias between scanners to a certain amount, leading to an increased accuracy of roughly the same magnitude as the pooled cross-validation: these findings suggest that our estimates of diagnostic accuracy can be generalized to new scanners that were not part of the training process.

### Limitations and Future Work

Our data set did not include data from subjects in prodromal stages of AD. Therefore, we could not evaluate performance of our approach in the prediction of AD dementia. Future work of the EDSD will extend the database to include subjects with MCI in order to investigate this topic. Moreover, we will investigate whether the DTI and MRI-derived indices provide complementary information regarding AD detection. This additional information could be exploited by the SVM classifier and improve the results.

### Conclusion

Our data suggest that machine learning algorithms together with multicenter DTI data provide a robust measure to assess white matter degeneration in AD dementia. The accuracy of our results compares favorably with earlier monocenter DTI studies. Cross-validation using the data from each scanner as an own fold suggest that our results can be generalized to new scanners. Future research will focus on early detection of AD specific structural WM changes in prodromal stages of AD. Presently, the EDSD study is collecting a multicenter DTI data set of MCI subjects that are characterized by CSF biomarkers and clinical follow-up.

## Supporting Information

Figure S1 **IG values of informative voxels for the group separation of AD and HC.** IG maps for (A) FA, (B) MD, (C) WMD, and (D) GMD. Clustered and thresholded at 0.05, these voxels define the mask for classification. The slices shown are: −46, −38, −28, −20, −10, −2, 8, 16, 26, 34 and 44 in MNI space.
(EPS)

Figure S2 **SVM sensitivity maps for (A) FA, (B) MD, (C) WMD, and (D) GMD.** The maps display the relative importance of each voxel for the classification decision, with white/yellow areas being more important than red areas. Preceding SVM classification, voxels that did not contribute any information to the group separation of AD and HC were masked out (IG criterion). The slices shown are: −46, −38, −28, −20, −10, −2, 8, 16, 26, 34, and 44 in MNI space.
(EPS)
